# Construction and internal validation of a novel nomogram for predicting prognosis of infective endocarditis

**DOI:** 10.1038/s41598-022-22173-5

**Published:** 2022-10-14

**Authors:** Zhao-Jun Yu, Zhi-Jie Ni, Jing Li, Guo-Xing Weng, Zhi Dou

**Affiliations:** 1 Department of Cardiovascular Surgery, Fujian Provincial Hospital, Fujian Medical University Provincial Clinical Medical College, No. 134, East Street, Fuzhou, 350001 Fujian Province China; 2 Shengli Clinical Medical College of Fujian Medical University, Fujian Medical University, Fuzhou, Fujian Province China

**Keywords:** Biomarkers, Cardiology, Diseases

## Abstract

To develop a nomogram prediction model capable of early identification of high-risk infective endocarditis (IE) patients. We retrospectively analyzed the clinical data of 383 patients with IE and divided them into survival and non-survival groups according to different hospitalization outcomes. Univariate and multivariate logistic regression methods were used to screen independent risk factors affecting the survival outcome of IE, and a Nomogram prediction model was constructed by these factors. The Hosmer–Lemeshow goodness-of-fit test was applied to assess the model fit, the discrimination and calibration of the model were evaluated by plotting ROC curves and calibration curves. Advanced age, embolic symptoms, abnormal leukocyte count, low hemoglobin level and double-sided IE were associated with higher in-hospital mortality in patients with IE (P < 0.05). The Hosmer–Lemeshow goodness-of-fit test for the model was χ^2^ = 7.107, *P* = 0.311. The AUC of the ROC curve of the model was 0.738 (95% CI 0.677–0.800). The bootstrap method was used to validate the prediction model. The results showed that the prediction accuracy of the model in the validation cohort was 0.842. The nomogram prediction model can accurately predict the in-hospital mortality risk of IE and can help clinicians identify high-risk IE patients early.

## Introduction

Infective endocarditis (IE) is a potentially fatal disease caused by bacteria, fungi and other microorganisms infecting the endocardium surface of the heart. The annual incidence of IE is low, ranging from 3 to 10 per 100,000 people^1^. Due to the devastating nature of the disease itself, patients with IE have a poor prognosis and often die from serious complications such as congestive heart failure, embolic events and sepsis. Despite great advances in treatment under current medical conditions, the in-hospital mortality rate of IE remains as high as 20%^2^. There are many factors influencing the prognosis of IE patients, including patient characteristics, the presence of cardiac and non-cardiac complications, the type of pathogenic microorganism, and echocardiographic findings^3^. Previous studies have found that early identification of high-risk IE patients and prompt medical and surgical treatment can significantly improve the prognosis of IE patients^4^.

Although clinicians have recognized that early identification of high-risk IE patients is critical for improving prognosis. However, compared to other common diseases in the clinic, IE lacks randomized controlled trials and a sufficient number of meta-analyses to screen for specific clinical features that indicate a poor prognosis^5^. Therefore, how to develop a tool that can accurately predict the prognosis of IE based on the early clinical features of patients is an urgent problem to be solved.

In recent years, clinical prediction models have become increasingly popular in the study of disease prognosis. Clinical predictive models use mathematical formulas to link disease risk factors to patient survival outcomes, and then use the model's probabilities to guide clinicians in their decisions. As a type of clinical prediction model, Nomogram transforms complex regression equations into simple and visual graphs, making clinical predictive models easy to use and understand even for the layman with no professional training^6^. In many studies, Nomogram prediction models have been shown to accurately predict outcome events and provide a basis for clinician decision making^7,8^.

We hope to develop a Nomogram prediction model based on early clinical features of patients to assess the risk of in-hospital death in IE. The probability predicted by the model can identify those high-risk patients with poor prognosis, and help clinicians formulate individualized management strategies for IE patients, ultimately achieving the goal of improving prognosis.

## Materials and methods

### Study population

We used the hospital electronic medical record system to collect clinical data on patients with IE who were diagnosed or suspected of having IE in our hospital from June 2012 to November 2021. A total of 694 cases were retrieved. The inclusion criteria for the study were patients who met the modified Duke diagnostic criteria for IE and the exclusion criteria were those subjects who did not meet modified Duke diagnostic criteria^9^. Finally, 383 study subjects were included, and the clinical data of the subjects was collected.

### Clinical data collection

We collected baseline information, history of previous cardiac disease, and history of risky diseases (maintenance hemodialysis, history of recent oral disease treatment, history of central venous cannulation, etc.) associated with IE from 383 study subjects. The collected laboratory results included blood routine, blood biochemistry, C-Reactive Protein (CRP), Procalcitonin (PCT), Brain Natriuretic Peptide (BNP), and blood bacterial culture at the early stage of the disease. The collected imaging data mainly includes Transthoracic Echocardiography (TTE) and Transesophageal Echocardiography (TEE). In order to reflect the early clinical features of patients with IE, the first examination after admission was used for patients who visited our hospital directly after the onset of the disease, and the examination results before receiving anti-infective drugs were used for patients who had been seen in other hospitals and were transferred to our hospital. The echocardiogram results shall be based on the first examination after admission, and if a patient has both TTE and TEE examinations, the TEE examination shall prevail.

### Definition of variables and outcome of the study

Embolic symptoms were defined as those associated with a recent stroke, pulmonary embolism, abdominal organ and extremity artery embolism. Vegetation was defined on cardiac echocardiography as a thrombotic mass with cluttered echoes and unstable motion independent of the valve^10^. Double-sided IE was defined as the presence of vegetation in both the left and right cardiac systems on ultrasound.

The outcomes of the study were divided into survival and non-survival groups according to the outcomes of IE patients after hospitalization.

### Variable screening and Nomogram prediction model construction and evaluation

First, the variables that may affect the outcome of the study were included in the univariate logistic regression analysis, and then the variables with statistical significance in the univariate regression analysis were included in the multivariate logistic regression analysis to obtain independent risk factors affecting the in-hospital mortality of IE patients. The nomogram prediction model was constructed using the screened independent risk factors. The Hosmer–Lemeshow goodness-of-fit test was used to evaluate the fit of the model, and the Receiver Operator Characteristic curve (ROC) was used to evaluate the predictive performance of the model. Then the bootstrap method was used to verify the prediction model, and a calibration curve was drawn to evaluate the consistency between the observation probability and prediction probability of the model. Finally, the potential clinical utility of the model was analyzed by decision curve and clinical impact curve.

### Statistical analysis

SPSS 26.0 statistical software and R (4.1.3) software were used for statistical analysis. The Kolmogorov–Smirnov and Shapiro–Wilk tests were used to determine whether the numerical variables were normally distributed. Continuous variables with normal distribution were expressed as mean ± standard deviation (x ± s), and independent samples t-test was used for comparison between groups. Non-normally distributed continuous variables were expressed as the median (interquartile range) M(IQR), and comparisons between groups were performed using the Mann–Whitney *U* test. Categorical variables were expressed as percentages, and comparisons between groups were performed using Pearson's chi-square test, continuous-adjusted chi-square test, or Fisher's exact test. *P* < 0.05 was considered statistically significant.

### Ethics approval and consent to participate

This study was reviewed and approved by the Ethics Committee of Fujian Provincial Hospital. All methods were carried out in accordance with relevant guidelines and regulations. Written informed consent to participate in this study was provided by the participants’ legal guardian/next of kin.

## Results

### Population characteristic

**Figure 1:**
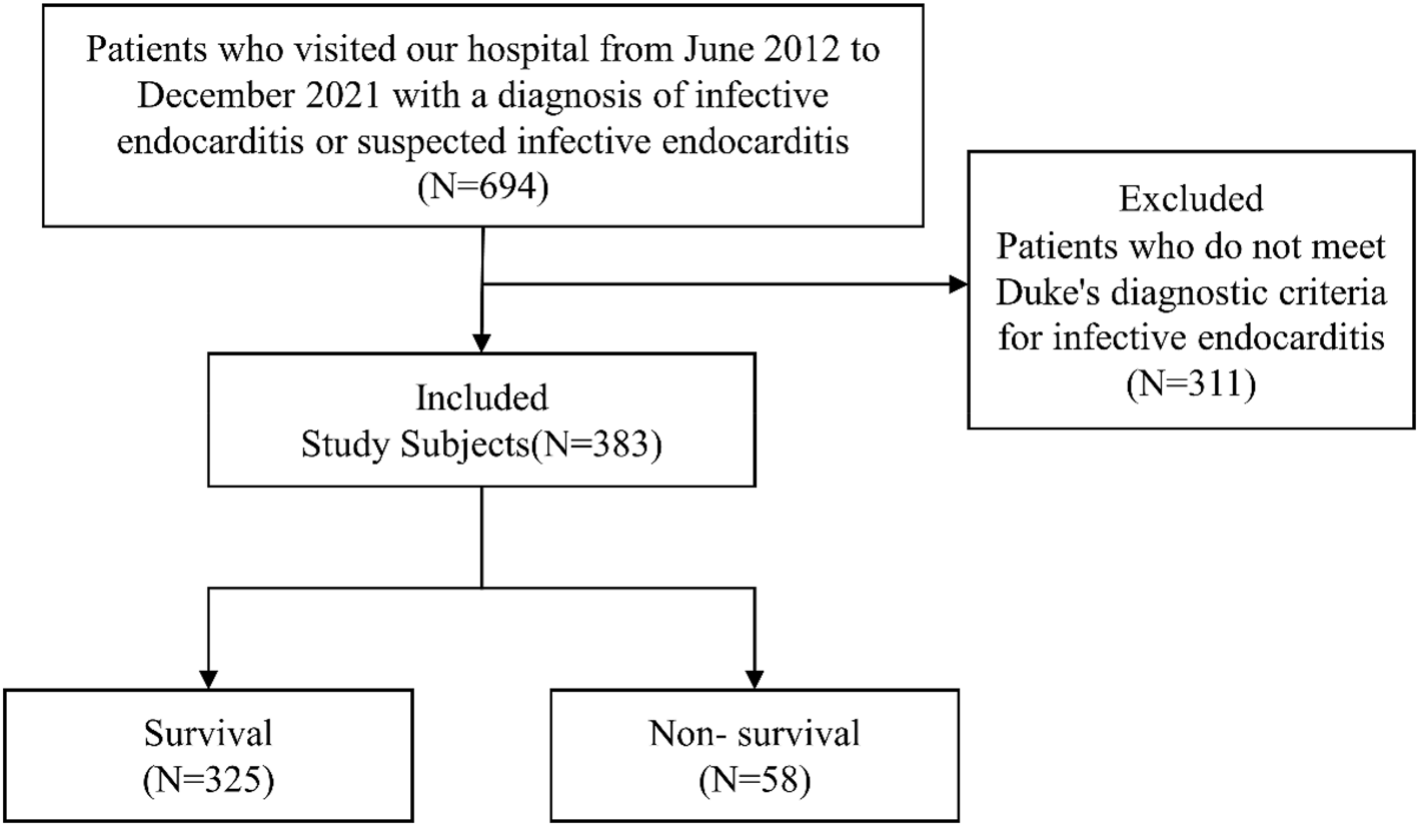
Study enrollment flowchart.

Following the inclusion and exclusion criteria of the study, we finally enrolled 383 patients with IE. Based on different study outcomes, we divided the enrolled study subjects into a survival group (N = 325) and a non-survival group (N = 58) (Fig. 1).

First, we analyzed the survival and non- survival groups' early clinical characteristics and laboratory data (Table 1). The age of patients in the survival group was (48.8 ± 16.3) years old, and that in the non-survival group was (58.8 ± 15.8) years old. There was a statistical difference in the overall mean age between the two groups (difference 10.00, 95% CI 5.45–14.56, t = 4.32, *P* < 0.001). The proportion of patients with embolic symptoms was 14.5% in the survival group and 27.6% in the non-survival group, and there was a statistical difference in the proportion of patients with embolic symptoms between the two groups (*P* = 0.013).

**Table 1:** Comparison of early clinical features and laboratory tests in the study population.

Variables	Overall (N = 383)	Survival (N = 325)	Non-survival (N = 58)	*P* value
Age (Mean ± SD)	50.36 ± 16.62	48.84 ± 16.33	58.84 ± 15.81	< 0.001
BMI (M, IQR)	21.88 (3.50)	21.63 (3.62)	22.15 (2.65)	0.446
**Gender, *n *(%)**				0.574
Female	120 (31.3)	100 (30.8)	20 (34.5)	
Male	263 (68.7)	225 (69.2)	38 (65.5)	
**Disease-process, *n *(%)**				0.255
Acute	96 (25.1)	78 (24.0)	18 (31.0)	
Non-acute	287 (74.9)	247 (76.0)	40 (69.0)	
**Clinical symptoms, *n *(%)**				
Fever				0.491
Yes	272 (71.0)	233 (71.7)	39 (67.2)	
No	111 (29.0)	92 (28.3)	19 (32.8)	
Embolic symptoms				0.013
Yes	63 (16.4)	47 (14.5)	16 (27.6)	
No	320 (83.6)	278 (85.5)	42 (72.4)	
Heart failure				0.050
Yes	240 (62.7)	197 (60.6)	43 (74.1)	
No	143 (37.3)	128 (39.4)	15 (25.9)	
**Past medical history**				
Valvular disease				0.141
Yes	108 (28.2)	87 (26.8)	21 (36.2)	
No	275 (71.8)	238 (73.2)	37 (63.8)	
Congenital heart disease				0.506
Yes	50 (13.1)	44 (13.5)	6 (10.3)	
No	333 (86.9)	281 (86.5))	52 (89.7)	
Prosthetic valve or intracardiac device				0.054
Yes	71 (18.5)	270 (83.1)	16 (27.6)	
No	312 (81.5)	55 (16.9)	42 (72.4)	
Maintenance hemodialysis				1.000
Yes	12 (3.1)	10 (3.1)	2 (3.4)	
No	371 (96.9)	315 (96.9)	56 (96.6)	
Central venous catheters inserted				0.287
Yes	19 (5.0)	14 (4.3)	5 (8.6)	
No	364 (95.0)	311 (95.7)	53 (91.4)	
**Laboratory tests (M, IQR)**				
Leukocyte count (× 10^9^/l)	9.53 (5.05)	9.20 (4.58)	10.99 (7.87)	0.006
Neutrophil count (× 10^9^/l)	7.10 (4.89)	6.99 (4.61)	8.68 (7.45)	0.007
PLT (× 10^9^/l)	203.00 (127.00)	210.00 (125.50)	182.52 (122.72)	0.007
CRP (mg/l)	48.52 (60.83)	45.90 (59.30)	68.61 (66.97)	0.008
PCT (ng/ml)	0.51 (6.21)	0.39 (6.08)	1.65 (8.12)	0.025
**Hemoglobin (g/l) (n, %)**				0.001
≥ 90	285 (74.4)	252 (77.5)	33 (56.9)	
< 90	98 (25.6)	73 (22.5)	25 (43.1)	
**Blood culture, *n* (%)**				
Positive	217 (56.7)	183 (56.3)	34 (58.6)	0.743
Gram positive	194 (50.7)	168 (51.7)	26 (44.8)	0.335
Gram negative	17 (4.4)	15 (4.6)	2 (3.4)	0.959
Fungus	6 (1.6)	0	6 (10.3)	< 0.001
**Echocardiography, *n *(%)**				
Valve vegetation				0.040
Yes	330 (86.4)	285 (87.7)	45 (77.6)	
No	52 (13.6)	40 (12.3)	13 (22.4)	
The size of the vegetation				0.272
< 10 mm	179 (46.7)	158 (48.6)	21 (36.2)	
≥ 10 mm	151 (39.4)	127 (39.1)	24 (41.4)	
Involved valve				
Mitral valve	212 (55.4)	181 (55.7)	31 (53.4)	0.751
Aortic valve	131 (34.2)	117 (36.0)	14 (24.1)	0.079
Tricuspid valve	21 (5.5)	16 (4.9)	5 (8.6)	0.409
Pulmonary valve	11 (2.9)	10 (3.1)	1 (1.7)	0.887
Multiple valves are involved	42 (11.0)	35 (10.8)	7 (12.1)	0.770
Double-sided IE	10 (2.6)	6 (1.8)	4 (6.9)	0.076

*BMI* Body Mass Index, *PLT* Platelet count, *CRP* C-reactive protein, *PCT* Procalcitonin, *IE* Infective endocarditis.

When laboratory tests were compared, we found that the median leukocyte count was 9.20 (4.58) × 10^9^/L in the survival group and 10.99 (7.87) × 10^9^/L in the non-survival group, with a statistically significant difference in the overall distribution of leukocyte count between the two groups (z = 2.74, *P* = 0.006). The percentage of patients with hemoglobin less than 90 g/L was 22.5% in the survival group and 43.1% in the non-survival group, and the difference in the percentage of patients with hemoglobin less than 90 g/L between the two groups was statistically significant (*P* = 0.001). When the cardiac ultrasonography results of the two groups were compared, we noticed that cardiac vegetations were present in 87.7% of the patients in the survival group and 77.6% of the patients in the non-survival group. There was a statistical difference in the proportion of patients with vegetation between the two groups (*P* = 0.040).

### Univariate and multivariate logistic regression analysis of risk factors

Risk factors that might affect study outcomes were included in univariate and multivariate logistic regression analyses (Table 2). Univariate logistic regression analysis revealed that age, embolic symptoms, abnormal leukocyte count, low hemoglobin level, and double-sided IE were associated with higher in-hospital mortality in patients with IE (*P* < 0.05). The above variables were included in the multivariate logistic regression analysis. Multivariate regression analysis showed age (> 50 years old) (OR 2.87, 95% CI 1.50–5.47, *P* = 0.001), embolic symptoms (OR 2.34, 95% CI 1.17–4.67, *P* = 0.016), abnormal leukocyte count (OR 2.11, 95% CI 1.15–3.87, *P* = 0.015), low hemoglobin (< 90 g/L) (OR 2.68, 95% CI 1.45–4.94, *P* = 0.002), double-sided IE (OR 6.11, 95% CI 1.49–25.07, *P* = 0.012) were independent risk factors for in-hospital mortality in patients with IE.

**Table 2:** Univariate and multivariate logistic regression analysis of risk factors for infective endocarditis.

Factors	Classification and description	Univariate analysis			Multivariate regression		
		OR	95% CI	*P*-value	OR	95% CI	*P*-value
Age	(≤ 50/ > 50)	2.38	1.31–4.32	0.004	2.87	1.50–5.47	0.001
Gender	(Female/male)	0.84	0.47–1.52	0.575			
BMI	(18.50–24.00/else)	0.57	0.29–1.09	0.089			
Disease-process	(non-acute/acute)	1.43	0.77–2.63	0.257			
**Clinical symptoms**							
Fever	(No/yes)	0.81	0.45–1.48	0.492			
Embolic symptoms	(No/yes)	2.25	1.17–4.33	0.015	2.34	1.17–4.67	0.016
Heart failure	(No/yes)	1.86	0.99–3.49	0.052			
**Past medical history**							
Valvular disease	(No/yes)	1.55	0.86–2.80	0.143			
Congenital heart disease	(No/yes)	0.74	0.30–1.82	0.507			
Prosthetic valve or intracardiac device	(No/yes)	1.87	0.98–3.56	0.057			
Maintenance hemodialysis	(No/yes)	1.13	0.24–5.27	0.881			
Central venous catheters inserted	(No/yes)	2.10	0.73–6.06	0.172			
**Laboratory tests**							
Leukocyte count (× 10^9^/l)	(4–10/else)	2.06	1.16–3.65	0.014	2.11	1.15–3.87	0.015
PLT (× 10^9^/l)	(100–300/else)	1.49	0.82–2.73	0.194			
CRP (mg/l)	(≤ 5.0 mg/l/> 5.0 mg/l)	2.00	0.59–6.77	0.264			
PCT (ng/ml)	(≤ 0.5 ng/ml/> 0.5 ng/ml)	1.77	1.00–3.15	0.050			
Hemoglobin (g/l)	(≥ 90/< 90)	2.62	1.46–4.68	0.001	2.68	1.45–4.94	0.002
Blood culture	(Negative/positive)	1.10	0.62–1.94	0.743			
**Echocardiography**							
The size of the vegetation (mm)	(< 10/≥ 10)	1.09	0.62–1.92	0.775			
Number of vegetation	(Single/multiple)	0.83	0.36–1.95	0.672			
**Involved valve**							
Mitral valve	(No/yes)	0.91	0.52–1.60	0.752			
Aortic valve	(No/yes)	0.57	0.30–1.08	0.082			
Tricuspid valve	(No/yes)	1.82	0.64–5.18	0.261			
Pulmonary valve	(No/yes)	0.55	0.07–4.40	0.575			
Multiple valves involved	(No/yes)	1.14	0.48–2.70	0.771			
Left-sided IE	(No/yes)	0.54	0.28–1.01	0.052			
Right-sided IE	(No/yes)	1.57	0.68–3.63	0.288			
Double-sided IE	(No/yes)	3.94	1.08–14.42	0.038	6.11	1.49–25.07	0.012

*OR* Odds Ratio, *CI* Confidence Interval, *PLT* Platelet count, *CRP* C-reactive protein, *PCT* Procalcitonin, *IE* Infective endocarditis.

### Construction and validation of a nomogram prediction model based on early clinical characteristics of patients with IE

**Figure 2:**
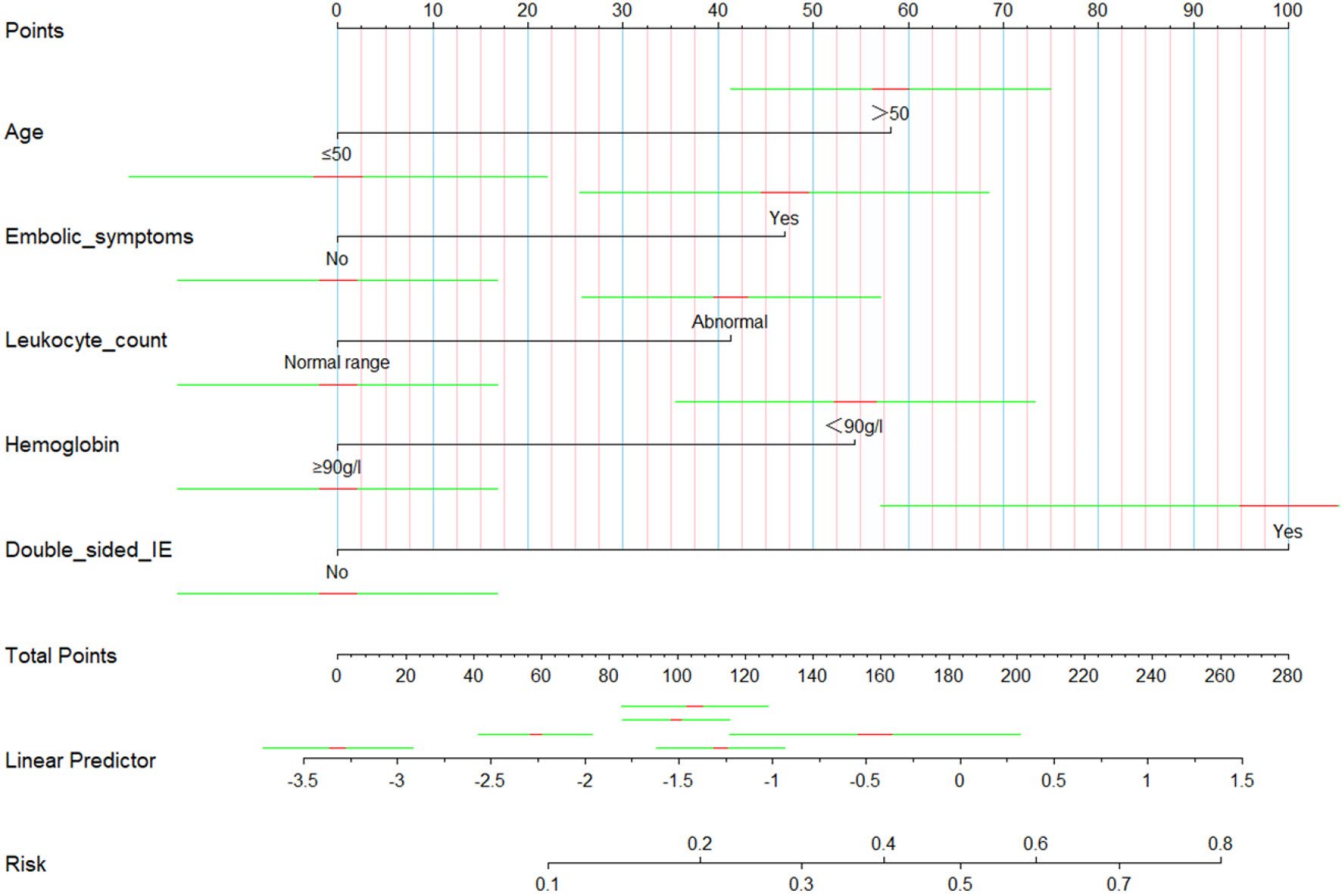
Nomogram for identification of high-risk patients with IE based on early clinical features. According to the early clinical features of patients, a straight line perpendicular to the points line was drawn to obtain the scores corresponding to different features of each variable, and the points of features of each variable were added up. Next, mark the sum on the total point axis and draw a straight line perpendicular to the risk axis. The red and green line segments represent the 10% and 70% confidence intervals of the scores corresponding to the variables.

**Figure 3:**
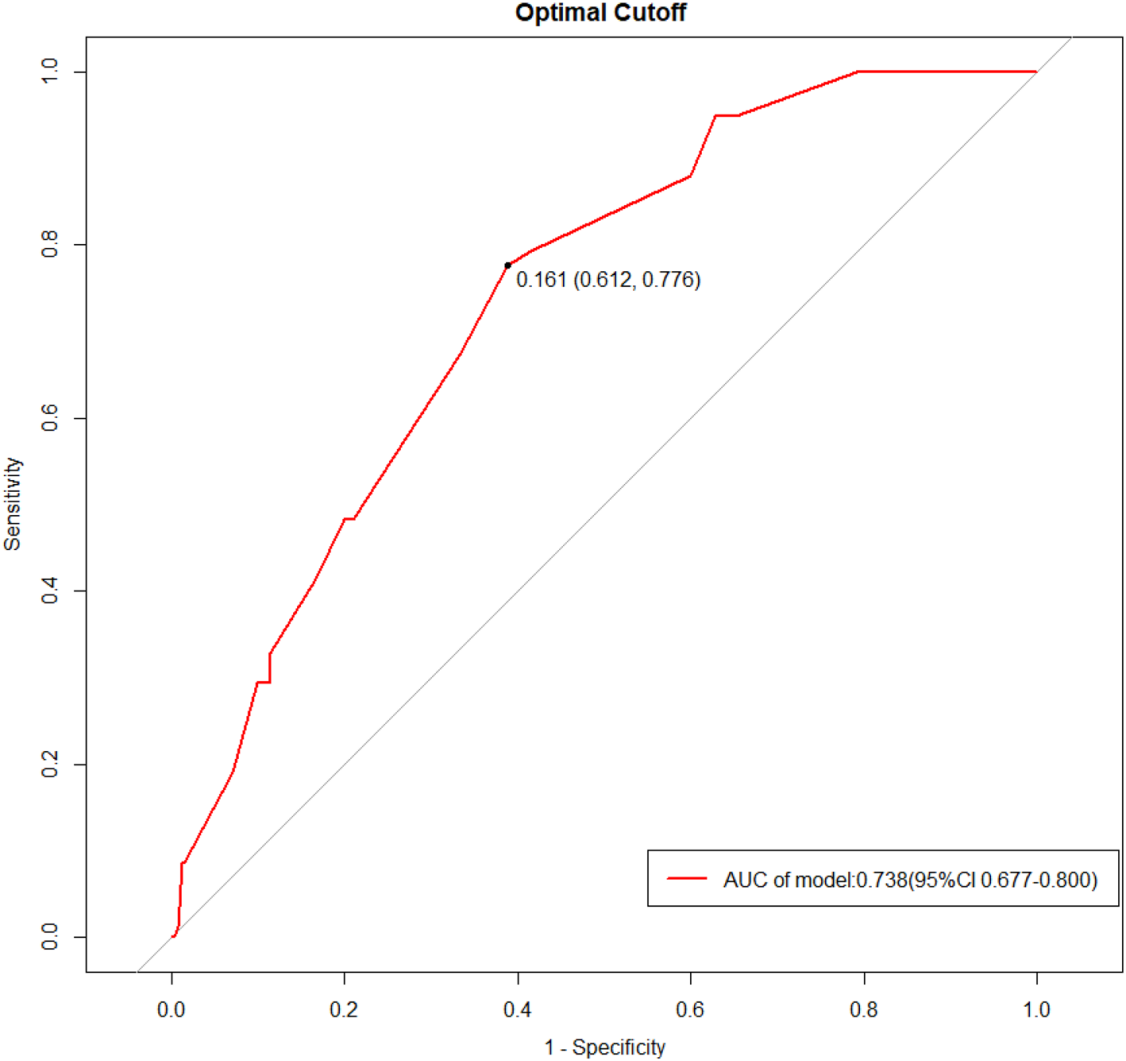
Receiver operating characteristic curve of the prediction model. The point in the upper left corner is the optimal cutoff value. *AUC* Area Under Curve.

**Figure 4:**
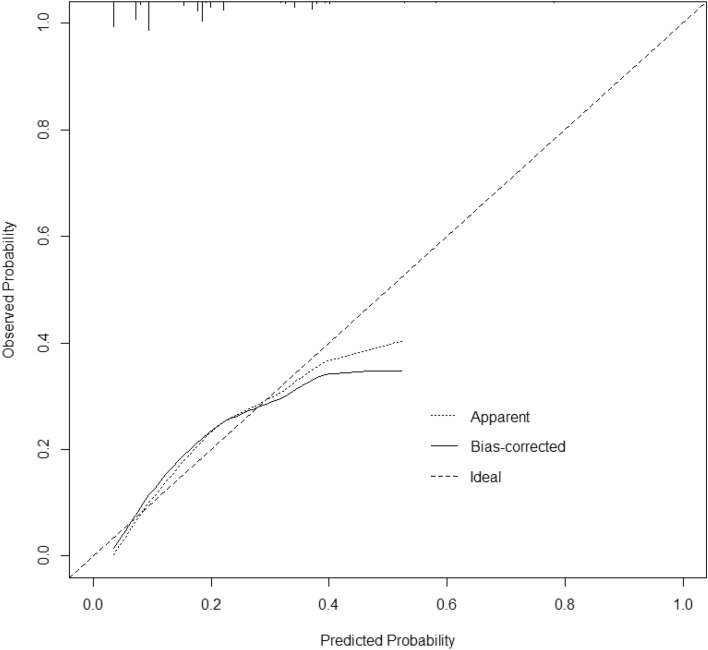
Calibration curve of the nomogram prediction model for IE. The x-axis depicts the predicted probability of dying during hospitalization, whereas the y-axis depicts the observed probability of dying during hospitalization.

The 5 variables screened out by multivariate logistic regression analysis were used to construct a model for predicting the risk of IE in-hospital mortality, and the model was displayed using a nomogram (Fig. 2). The Hosmer–Lemeshow goodness-of-fit test for the model was χ^2^ = 7.107, *P* = 0.311, indicating a good model fit. The model's predictive ability was evaluated using the ROC curve, which revealed an area under the curve (AUC) of 0.738 (95% CI 0.677–0.800) (Fig. 3). The optimal cutoff value of the ROC curve was 0.161, and at this threshold, the sensitivity of the model was 77.6% and the specificity was 61.2%. The prediction model was validated using the bootstrap method, and the results showed that the model prediction accuracy was 0.842, indicating that it has excellent prediction capability. A calibration curve was created using R software to further evaluate the model's validity (Fig. 4), which indicates that the model's calibration curve fits well with the standard ideal curve.

### Clinical application of the Nomogram prediction model

**Figure 5:**
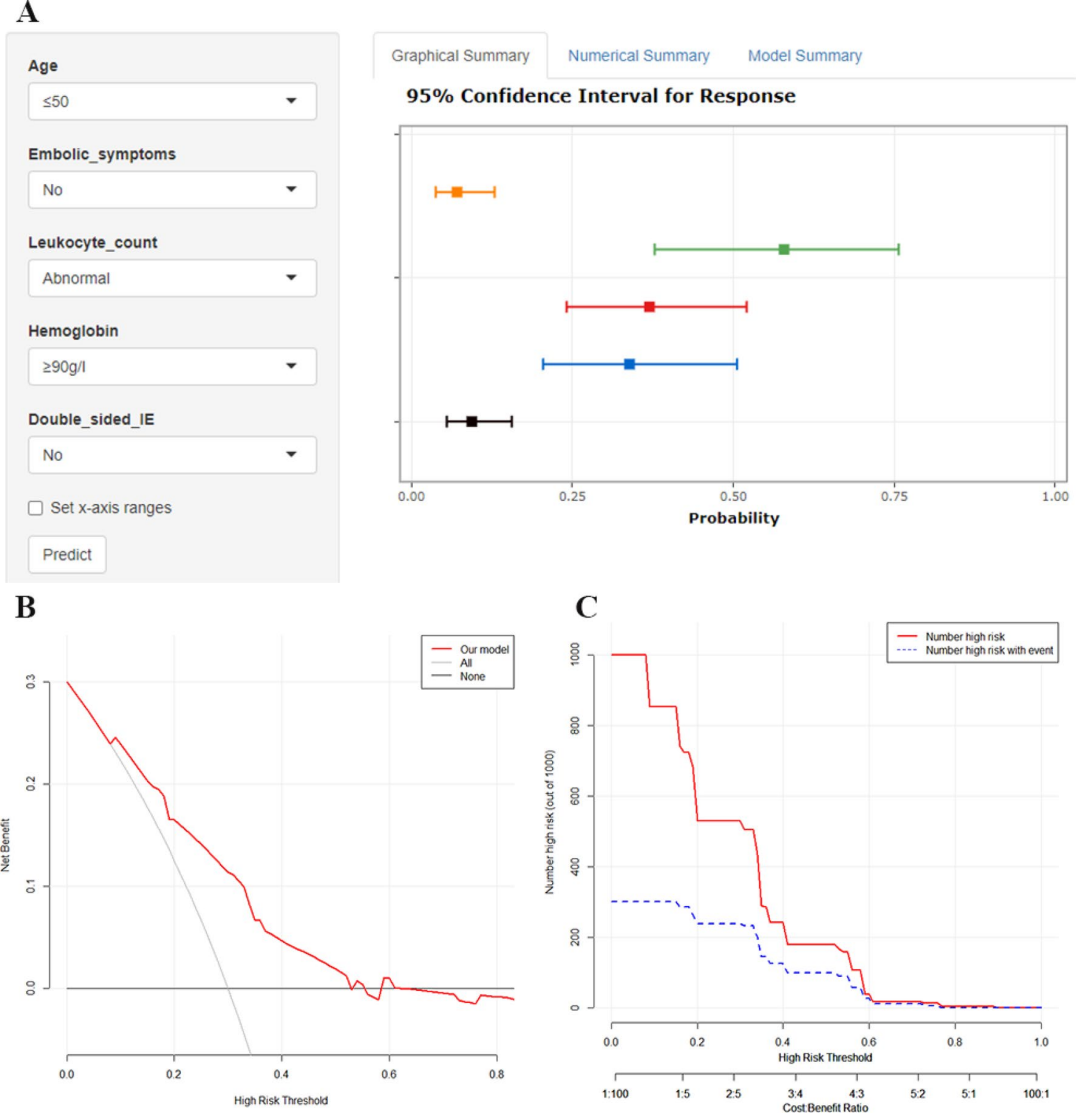
(**A**) Dynamic nomogram of the model, the dynamic nomogram will instantly calculate the in-hospital mortality rate for patients with IE by entering the patient's early clinical parameters. (**B**) Decision curve analysis of Nomogram prediction model, the X-axis indicates the threshold probability of an in-hospital death outcome, the Y-axis measures the net benefit. The gray line represents the assumption that all subjects had positive events (in-hospital deaths), the black line represents the assumption that all subjects had no positive events, and the red line represents the net benefit of the intervention at different threshold probabilities. (**C**) Clinical impact curves of the Nomogram prediction model. Applying the model to predict risk stratification for 1000 individuals, the red line indicates the number of people classified as positive (high risk) by the model at each threshold probability; the blue curve (Number high risk with outcome) is the number of true positives at each threshold probability.

We created a dynamic nomogram using the shiny package in R software to make our developed nomogram easier to use in clinical practice (Fig. 5). Visit the following URL to use our dynamic nomogram (https://yuzhaojun.shinyapps.io/DynNomapp/). The potential clinical utility of the model was analyzed by the decision curve and clinical impact curve. Using the decision curve, we found that at a threshold probability of 10–50%, the net benefit of classifying patients at high risk of IE using the probability predicted by Nomogram would be higher than that of treating all or nothing. Using the optimal cut-off value of ROC curve 0.161 as the threshold probability, the net benefit rate is higher than 20% and the cost–benefit ratio is about 1:5.

## Discussion

In this study, we used 383 IE patients as the research subject and developed a Nomogram prediction model that can predict IE patients' in-hospital mortality. By univariate and multivariate logistic regression analysis, we found that advanced age, embolic symptoms, abnormal leukocyte count, low hemoglobin level, double-sided IE were independent risk factors for in-hospital mortality in patients with IE. Based on the above five independent risk factors, we developed a clinical prediction model, and display it with a Nomogram. The Hosmer–Lemeshow goodness-of-fit test of the model showed χ^2^ = 7.107, *P* = 0.311, indicating a good model fit. The model's predictive performance was evaluated by drawing the ROC curve, and the result indicates that the AUC was 0.738. (95% CI 0.677–0.800). The bootstrap approach was used to validate the prediction model, and the results showed that the model prediction accuracy was 0.842, showing that our established prediction model has good prediction accuracy in the validation queue as well.

We created a dynamic nomogram on the web page to make the nomogram prediction model more user-friendly. The dynamic nomogram can quickly calculate the in-hospital mortality of IE patients by inputting their early clinical features. When we use the optimal cutoff value of 16.1% on the ROC curve as the threshold probability (above which the model predicts the probability of being a high-risk patient and below which the model predicts the probability of being a low-risk patient), the risk stratification of patients by the model's predicted probability results in a net benefit rate of more than 20% and a cost–benefit ratio of about 1:5.

It was found that higher levels of inflammatory markers (leukocyte count, CRP, PCT) on admission were associated with higher in-hospital mortality in patients with IE^11^. In our study, abnormal leukocyte count (OR 2.11) was also associated with poor prognosis in patients with IE. In patients with severe infections, bone marrow suppression and low hematopoietic capacity may occur, so low hemoglobin level (OR 2.68) is also associated with higher in-hospital mortality in IE patients. Advanced age was also an independent risk factor for in-hospital death in patients with IE, with patients older than 50 years having a significantly higher risk of in-hospital death than those younger than 50 years (OR 2.87). Patients with embolic symptoms had a significantly increased risk of in-hospital death (OR 2.34), experts currently believe that embolic symptoms like stroke are prevalent and life-threatening complications for patients with IE^3,12,13^. In contrast to the traditional belief that IE patients require a sufficient course of anti-infective treatment prior to surgery, an increasing number of clinicians believe that in IE patients with cardiac vegetations, early surgery not only removes the lesions and restores normal cardiac function, but also reduces the patient's risk of embolic events^14^. Double-sided IE (OR 6.11) often means that the normal function of both the left and right cardiac systems of the patient is compromised, which not only increases the likelihood of congestive heart failure, but also represents a much higher risk of embolic events than patients with left-sided IE or right-sided IE.

In recent years, as risk factors closely related to IE prognosis have been identified, many clinicians have constructed clinical prediction models by searching for risk factors that affect the survival outcome of IE patients. In 2003, Hasbun et al. first established a model capable of predicting the 6-month risk of death in IE patients. The model incorporated five risk factors: Carlson comorbidity score, abnormal mental status, moderate to severe congestive heart failure, bacterial etiology other than viridans streptococci, and absence of surgical treatment. Each risk factor had a corresponding scoring system, and patients were classified into four risk groups according to their scores, with 6-month mortality rates of 5%, 15%, 31%, and 59%, respectively^13^. In our study, the risk factors included in the analysis also consisted mainly of patients' baseline data, underlying disease and related complications caused by IE. But compared with Hasbun's model, our model has advantages in the following aspects. First, there is no scoring system that requires subjective assessment by clinicians, and our model incorporates more realistic, objective laboratory tests (such as blood test results). Second, our model is applicable to all patients with IE, whereas Hasbun's model is only applicable to patients with complicated left-sided native valve endocarditis. Third, although the model developed by Hasbun has used a more concise scoring system, this still requires clinicians to calculate scores for each risk factor and then sum them to obtain the probability of risk for patients with IE. However, by using our dynamic nomogram, users only need to enter the characteristics of each risk factor on the web page, the model will immediately output the risk probability of in-hospital death in IE patients.

After the first model for predicting the mortality of IE patients was developed, more and more clinicians have constructed clinical prediction models based on the risk factors affecting the prognosis of IE patients. In 2011, Sy et al. developed a time-dependent risk prediction model for IE by retrospectively analyzing the clinical data of 273 IE patients. The model incorporated clinical features of IE patients on days 1, 8, and 15 of hospitalization, and then, these clinical features were used as key predictors to develop 3 independent risk prediction models. The prediction accuracies of the three independent models were 0.79, 0.79 and 0.84, respectively^15^. Sy's risk prediction model incorporates many variables such as age, tachycardia, embolic events, renal impairment, heart failure, thrombocytopenia, and severe comorbidities. Sy's study confirmed that the prognosis of patients with IE can be accurately predicted by the clinical features of the patients. However, a predictive model that includes too many variables not only may overfit, but also complicate the use of the model. We believe that the ultimate purpose of building a clinical predictive model is to make it clinically applicable. After fully considering the early clinical characteristics of IE patients, the Nomogram predictive model we developed included only 5 variables and performed well in predicting the prognosis of IE patients.

As the Nomogram has gained increasing attention in the clinic, the Nomogram prediction model has also been applied to the prognostic study of IE. 2021 Li et al. developed a Nomogram based on the neutrophil-to-platelet ratio (NPR) to predict in-hospital mortality in patients with IE, and risk stratified IE patients by their NPR at admission to the hospital. The AUC of this nomogram prediction model ROC was evaluated to be 0.742, which confirmed the good prediction performance of the model^16^. Through Li's study, we found that the Nomogram prediction model performed well in risk stratification of IE patients. However, Li's model incorporates only NPR as a basis for risk stratification of IE, which cannot fully reflect the clinical characteristics of IE. The model we developed comprehensively considered the early clinical features, laboratory tests and echocardiographic findings of IE, and more comprehensively reflected the early clinical features of patients. Furthermore, the Nomogram prediction model we created is more user-friendly and more suited to widespread adoption and application in clinical practice.

## Limitation

First, this is a retrospective study, and retrospective analyses are inevitably subject to data loss and case selection bias. Second, this was a single-center study that could not accurately represent the epidemiological and clinical characteristics of all patients with IE in the region. Therefore, in the future, there is a necessity to include patients with IE from more regions and to build a multicenter source of data to construct a better clinical prediction model.

## Conclusion

In conclusion, we found that age (> 50 years old), embolic symptoms, abnormal leukocyte count, low hemoglobin level (< 90 g/L), and double-sided IE were independent risk factors for the incidence of in-hospital death in patients with IE. The Nomogram prediction model based on early clinical features of patients can accurately predict the risk of in-hospital death in IE patients. Compared with traditional clinical prediction models, the Nomogram prediction model we developed is more convenient and more conducive to promotion and clinical application.

## Data Availability

The data of the current study are available from the corresponding author upon reasonable request.

## Abbreviations

IE: Infective Endocarditis
BMI: Body Mass Index
CRP: C-Reactive Protein
PCT: Procalcitonin
BNP: Brain Natriuretic Peptide
PLT: Platelet
TTE: Transthoracic Echocardiography
TEE: Transesophageal Echocardiogram
HF: Heart Failure
ROC: Receiver Operator Characteristic Curve
AUC: Area Under the Curve
IQR: Inter Quartile Range
CHD: Congenital Heart Disease
OR: Odds Ratio
